# Inter‐rater reliability of the Team Formulation Quality Rating Scale (TFQS) in inpatient mental health teams

**DOI:** 10.1111/bjc.70028

**Published:** 2026-01-02

**Authors:** Joshua Eli Thompson, Gillian Haddock, Georgia Penn, Katherine Berry

**Affiliations:** ^1^ Division of Psychology and Mental Health, School of Health Sciences, Faculty of Biology, Medicine and Health, Manchester Academic Health Science Centre University of Manchester Manchester UK; ^2^ Pennine Care NHS Foundation Trust Ashton‐Under‐Lyne UK; ^3^ Greater Manchester NHS Foundation Trust, Research and Innovation, Manchester Royal Infirmary Manchester UK

**Keywords:** measure, psychological mindedness, psychometric, reliability, scale, team formulation

## Abstract

**Objectives:**

Team formulation is associated with better working relationships between staff and service users. However, there is a need for greater standardization of practice. We aimed to investigate the inter‐rater reliability of the Team Formulation Quality Rating Scale (TFQS) and explore what aspects of team formulation practices were most frequently adhered to.

**Method:**

Staff at nine acute mental health wards participated in team formulation sessions facilitated by Health Care Professions Council registered psychologists. Formulation sessions were audio recorded, and raters used recordings to complete the TFQS. At least two raters rated 19 team formulation sessions.

**Results:**

The TFQS demonstrated excellent inter‐reliability for the total scale (ICC = .926, 95% CI = .820 to .971) and moderate inter‐rater reliability for subsection A (ICC = .660; 95% CI = .278 to .862) and subsection B (ICC = .733; 95% CI = .361 to .898). Overall, the items for ‘collaboration’ and ‘consideration of life events’ were rated better in terms of quality, compared with items relating to ‘close of meeting’ and ‘consideration of goals and values’ which tended to receive lower quality ratings.

**Conclusion:**

The TFQS is a reliable tool for measuring quality of team formulation within inpatient settings and should be used in future research and clinical practice. Psychometric properties should be assessed across different clinical settings. Training and supervision should ensure that psychologists' formulations incorporate a focus on the individual's goals and values impacting problem development and resolution.

**Practitioner points**

- Team formulation is recommended practice for psychologists in mental health settings, however, there is need for greater standardization of team formulation practices.
- The Team Formulation Quality Rating Scale (TFQS) provides a method of evaluating clinicians' performance in developing and facilitating a team formulation.
- The study found excellent inter‐rater reliability for the full scale TFQS, indicating that the TFQS is a reliable tool for measuring the quality of team formulation sessions in acute mental health settings.
- The study indicated the need for further consideration of the person's goals and values, strengths and social and cultural factors within team formulation practices.

## INTRODUCTION

Psychological formulation has been defined as ‘a hypothesis about a person's difficulties, which links theory with practice and guides the intervention’ (British Psychological Society Division of Clinical Psychology (DCP), 2011, p. 2). It is seen as a key process in explaining the development and maintenance of a person's difficulties and has been viewed as complementary to or as an alternative to psychiatric diagnosis (Johnson & Boyle, 2018; Seery et al., 2021). The ability to construct formulations of clients' difficulties is a ‘core competency’ of clinical psychologists and is a training requirement of other mental health professions (Association of Clinical Psychologists (ACP), 2022; DCP, 2011; Royal College of Psychiatrists, 2010). Formulation practices are increasingly being used within multidisciplinary team settings within mental health and social care (ACP, 2022). A ‘team formulation’ is typically facilitated by psychologists and draws on knowledge from multiple clinicians in a team to construct a collaborative understanding about a service user's difficulties, needs, and goals (ACP, 2022). A team formulation can be informed by one or multiple psychological models including cognitive behavioural (CBT), CBT‐interpersonal, cognitive analytic, power threat meaning framework, and 5p's approaches (Berry & Hartwell, 2023; Geach et al., 2018; Hartley, 2021; Nikopaschos et al., 2023). Rationales for team formulation include improving staff empathy towards service users, informing support plans, and developing more consistent ways of working (ACP, 2022; DCP, 2011). Three systematic reviews of team formulation practices have incorporated studies from a range of settings using both qualitative and quantitative methods to evaluate practice (Bealey et al., 2021; Geach et al., 2018; Short et al., 2019). The reviews report promising evidence relating to service user and staff member outcomes following team formulation. Staff teams reported increased knowledge and understanding of service users within the team, increases in positive attitudes and compassion towards service users, and increased perception of effectiveness, communication, and team working (Bealey et al., 2021; Geach et al., 2018; Short et al., 2019). Staff members reported feeling listened to by the facilitator and felt that team formulation led to changes in the team's thinking regarding their approach to supporting service users (Bealey et al., 2021). Service users have reported an improved working alliance with staff, improved staff understanding of their needs, improved ward organization, and reductions in perceptions of being criticized by staff following the team's participation in team formulation (Berry et al., 2015, 2017). However, the reviews of team formulation have indicated variable quality of research and limited use of validated outcome measures (Geach et al., 2018; Short et al., 2019). Whilst these reviews have summarized the aims and outcomes associated with team formulation, quantitative research regarding the content of team formulation practices is limited. Therefore, the degree to which facilitators of team formulation practices have adhered to processes of team formulation is not well reported (Geach et al., 2018). This lack of specificity may have contributed to team formulation being used as a ‘catch all’ term to describe a range of different practices (Geach et al., 2018). Consequently, the review by Geach et al. (2018) indicated a need for greater specification and standardization of team formulation practices. This development would enable clearer understanding of outcomes associated with team formulation and learning regarding best practice.

Research exploring individual clinicians' skill in case formulation has indicated that clinicians' self‐reported fidelity to models of case formulation may be unreliable. Zivor et al. (2013) reported a gap between therapists' perceived competency and actual performance in developing cognitive‐behavioural case formulation. In a study by Kuyken et al. (2005) it was reported that only 44% of cognitive‐behavioural case formulations developed by clinicians were rated as ‘at least good enough’ when compared with a benchmark case formulation, though it was noted that this proportion increased in a sub‐group accredited to use a cognitive‐behavioural approach in their practice. Research has also indicated that therapists may overestimate their fidelity to and implementation of various therapeutic models (Carroll et al., 1998; Creed et al., 2014; Hogue et al., 2013; Hurlbert et al., 2010; Martino et al., 2009). However, similar research regarding fidelity and quality of practices in team formulation settings is lacking. To carry out such research, it is necessary to have a standardized tool to measure the quality of team formulation practices. The Team Formulation Quality Rating Scale (TFQS; Bucci et al., 2021) was developed to provide a method of evaluating clinicians' performance in developing and facilitating a team formulation. The authors aimed to create a tool that was founded on evidence‐based models of formulation whilst capturing core elements of team formulation that are applicable across multiple models of understanding and intervention (Bucci et al., 2021). The scale is based on the intervention manual for delivering case formulations with mental health teams (Berry et al., 2015), and development of the tool was informed by the Motivational Interviewing‐Cognitive Behaviour Therapy Scale (Mi‐CBT; Haddock et al., 2012) and the Collaborative Case Conceptualization Rating Scale (CCCRS; Padesky et al., 2011). The tool consists of two sections assessing the structure and content of team formulation practices. The authors reported high content and face validity indicating that the tool and its subcomponents rate underlying constructs relevant to team formulation (Bucci et al., 2021). Inter‐rater reliability was evaluated in the original study between two researchers who used the TFQS to rate eight team formulation sessions carried out with mental health rehabilitation teams. A moderate level of inter‐rater reliability was reported for the full scale (ICC = .585; 95% CI = −1.675 to .920) and for Section A (structure; ICC = .527; 95% CI = –.253 to .813). A good level of inter‐rater reliability was reported for Section B (content; ICC = .785; 95% CI = –.208 to .958). Whilst the level of reliability for the full scale appears somewhat modest, it is generally consistent with other measures of psychological formulation quality (Bucci et al., 2016). The authors also reported acceptable internal consistency of the total scale but questionable internal consistency for the subscales.

The TFQS relies on observer ratings rather than facilitators' own ratings of team formulations (Bucci et al., 2021). However, it is possible that the TFQS may also be used by clinicians and trainee psychologists as a self‐assessment tool. The importance of the distinction in the views of those involved in team formulation, for example participants and facilitators, has been identified as an important factor in the development of the evidence base regarding team formulation (Bealey et al., 2021). Exploring differences between independent ratings and team formulation facilitators' own ratings of team formulation quality using the TFQS would inform the use of the TFQS as a self‐assessment tool for clinicians and trainee psychologists aiming to evaluate their own team formulation practice.

The current study had two main aims. (1) First, the study aimed to evaluate the reliability of the TFQS in measuring the quality of team formulation sessions outside of the original scale development study and with a larger sample size. (2) The study also aimed to investigate what aspects of team formulation practices in terms of content and processes were most frequently adhered to in mental health settings as measured by individual items of the TFQS. A third subsidiary aim was to explore the relationship between independent ratings and team formulation facilitators' own ratings of team formulation quality using the TFQS.

## METHOD

### Design

The current study analysed audio recordings of team formulation sessions, which were collected as part of a randomized controlled trial of a psychological intervention based on acute mental health wards. The Talk, Understand, Listen for Inpatient Settings (TULIPS) trial recruited 34 acute inpatient mental health wards from NHS foundation trusts in the United Kingdom (Berry et al., 2022). Seventeen wards were randomly allocated to receive an intervention that aimed to increase access to psychologically informed care and therapies on acute mental health wards (Berry et al., 2022). Wards included both male and female acute inpatient wards. Team formulation was a key element of the intervention, and all staff working in wards in the intervention arm of the trial were invited to team formulation sessions.

### Participants

Team formulation sessions were facilitated by six independent health care professions council registered psychologists (4 = White British, 6 = female) working across nine acute mental health wards. Psychologists reported an average of 6.6 years of qualified psychological practice at the time of the TULIPS trial. In addition to their core professional training in formulation, psychologists received one and a half days of training in team formulation, which was delivered by an author of the TFQS (KB).

Staff that attended team formulation sessions included registered mental health nurses, nursing assistants, activity co‐ordinators, occupational therapists, and psychiatrists from the 17 acute mental health wards allocated to the intervention arm of the trial. Details regarding the profession and demographics of healthcare staff attending each team formulation session were not collected.

### Measures

#### Team formulation quality rating scale (TFQS)

The TFQS is an observer‐rated measure of the quality of a team psychological formulation (Bucci et al., 2021). The scale comprises 15 items over two sections pertaining to the *structure* and *content* of the team formulation being evaluated. A three‐point rating scale is used to indicate the presence of each item measured by the scale, with items allocated a score of 2 (yes), 1 (to some extent), or 0 (no). Scores for the seven items in Section A (*structure*) and eight items in Section B (*content*) are summed to provide an overall score (*Section A* maximum = 14; *Section B* maximum = 16; *total* maximum score = 30) indicating the quality of the team formulation. Bucci et al. (2021) reported that the scale has demonstrated adequate content validity and face validity with expert consultation in the development. Acceptable internal consistency (α = .718) and a moderate level of inter‐rater reliability were also reported for the full scale (ICC = .585; 95% CI = −1.675 to .920), moderate reliability for Section A (ICC = .527; 95% CI = –.253 to .813), and good reliability for Section B (ICC = .785; 95% CI = –.208 to −.958).

#### Self‐reported formulation quality

Psychologists recruited to the TULIPS trial completed a diary to record their activity on the wards within the study. As part of this diary, psychologists submitted details relating to the formulation, including the date, ward, duration, and psychological approaches used in the formulation. Psychologists also completed and submitted an adapted version of the TFQS following each team formulation session. The adapted version of the TFQS included all items of the standard TFQS but used an adapted dichotomous (‘yes’, ‘no’) scoring system to indicate the presence of factors associated with each item, with items separated into sub‐items. For example, when using the standard TFQS, the item ‘key problems and needs are elicited’ is measured on a three‐point scale based on (1) whether the service user's needs are elicited and the needs are well operationalized in the formulation, and (2) whether differences between the needs of the service user and the needs or perceptions of staff are explored. For ease of rating, these two points were scored as separate items on the adapted version of the TFQS using the dichotomous scoring system. In total, the adapted measure incorporated 30 items, which are scored using the same dichotomous item. Both the standard and adapted versions of the TFQS had a maximum score of 30 for each formulation.

### Procedure

Ethical approval for the TULIPS randomized controlled trial was obtained from the Greater Manchester NHS Research Ethics Committee in July 2019 (IRAS ID: 264686). Approval included the use of data obtained in secondary research studies, and participants in the research trial provided informed consent for the use of data in secondary research.

Team formulation sessions were facilitated and audio recorded by psychologists using an encrypted device, with recordings stored on a secure drive. A total of 97 team formulation sessions were reported by psychologists from nine acute mental health wards. Of these, 48 team formulation sessions (49.5%) were audio recorded and used in the analysis. Not all formulation sessions were audio recorded as the study protocol stated that all staff present during the meeting need to give written informed consent for the session to be recorded and used in the research. One psychologist facilitated 29 (60.4%) team formulation sessions, with the remaining 19 formulation sessions distributed evenly between the remaining five psychologists. Incomplete formulation sessions recordings (*n* = 2) were excluded from the analysis.

The quality of team formulations was assessed by the first author (JT) using the TFQS following training in the use of the TFQS by an original author of the scale (KB). Evidence pertaining to each item of the TFQS was noted and inputted into a scoring sheet whilst listening to recordings of team formulation sessions. Each item of the TFQS was rated after reviewing the full audio recording, and a total TFQS score was calculated for each team formulation session. A senior clinical psychologist with experience in psychological formulation in mental health settings was recruited and trained in the use of the TFQS. Both the first author and the clinical psychologist recruited demonstrated good inter‐rater reliability with the scale author (KB) prior to administering the TFQS (ICC = .715; 95% CI = .242 to .949). Throughout the rating period, the first author (JT) and the senior clinical psychologist met regularly with the scale's author (KB) for supervision together to check their understanding of items and to receive additional training where necessary. The recruited clinical psychologist independently administered the TFQS to 40% of the recorded team formulation sessions using the same method as the first author described above.

### Data analysis

All data analysis was carried out in IBM SPSS for Mac version 28. Descriptive data were generated for the average duration of team formulation sessions and the frequency of models adopted in formulation. Data were extracted to assess the frequency of all individual items measured by the TFQS across all formulations. A two‐way mixed effects intraclass correlation coefficient of absolute agreement was calculated to investigate the inter‐rater reliability between the primary author (JT) and the clinical psychologist's ratings of the full scale TFQS, Section A, and Section B of the TFQS. Cohen's kappa was calculated to investigate the inter‐rater reliability of individual items of the TFQS.

A Pearson's correlation was also conducted to investigate the relationship between the researcher's scoring of formulations using the TFQS and the ward psychologists' ratings using the adapted version of the TFQS. There were several cases relating to team formulation sessions in which psychologists did not report a score for individual items of the adapted TFQS (reasons unknown). Missing data were excluded from the analysis.

## RESULTS

### Descriptive statistics

The quality of 46 team formulation sessions was measured using the TFQS (*M* = 17.48, SD = 3.60, Range = 8–25). Psychologists' own ratings of team formulation quality using the TFQS were also summarized (*n =* 83; *M =* 22.83; SD = 3.82; Range = 13–29). The reported duration of formulation sessions was 60 min on average (Range = 30–120). Various approaches to the formulation sessions were reported, including compassion focused (*n* = 26 [54.2%]), cognitive behavioural (*n* = 7 [14.6%]), 5P's framework (*n* = 4 [8.3%]), mixed (*n* = 2 [4.2%]), and power threat meaning framework (*n* = [2.1%]). The approach was not reported for eight formulation sessions. The number of ward staff members in each formulation session was not recorded but was typically between three and six.

### Aim 1: Inter‐rater reliability of the Team Formulation Quality Rating Scale (TFQS)

Two‐way mixed effects intraclass correlation coefficient model of absolute agreement indicated excellent inter‐rater reliability between the raters using the total TFQS scale (ICC = .926, 95% CI = .820 to .971), moderate inter‐rater reliability for Section A (ICC = .660; 95% CI = .278 to .862), and moderate inter‐rater reliability for Section B of the TFQS (ICC = .733; 95% CI = .361 to .898). Cohen's kappa analyses indicated slight to substantial agreement between the raters for individual TFQS items (range: *k =* .212 to .838). The results of individual analyses were statistically significant for all items apart from the items ‘goals and values’ and ‘team coping’. The results for the reliability of individual items are presented in Table 1.

**TABLE 1:** Results of reliability analyses for individual TFQS items.

Item	Outcome of inter‐rater reliability analysis
A1_Session Opening	*k* = .717; *p* < .001
A2_Collaboration	*k* = .469; *p* = .005
A3_Interpersonal Effectiveness	*k* = .628; *p* = .009
A4_Eliciting and responding to feedback	*k* = .393; *p* = .018
A5_Summary Statements	*k* = .364; *p* = .050
A6_Pacing	*k* = .407; *p* = .024
A7_Close of meeting	*k* = .414; *p* = .038
Section A Total	ICC = .660; *p* = .002
B1_Description of service user	*k* = .528; *p* = .013
B2_Problems	*k* = .579; *p* = .006
B3_Strengths	*k* = .617; *p* < .001
B4_Goals and Values	*k* = .378; *p* = .064
B5_Life events	*k* = .673; *p* = .006
B6_Team Coping	*k* = .212; *p* = .309
B7_Social and cultural factors	*k* = .398; *p* = .008
B8_Support plans	*k* = .838; *p* < .001
Section B Total	ICC = .733; *p* < .001

Abbreviations: ICC, intraclass correlation coefficient; *k*, Cohen's kappa.

### Aim 2: Team formulation quality and content

Frequency of scoring and mean scoring for each TFQS item can be found in Table 2. The mean rating across all team formulation sessions evaluated is presented in the table along with frequencies of ratings of ‘yes (score of 2)’, ‘to some extent (1)’ and ‘no (0)’ consideration of that item within the team formulation session. Regarding team formulation session structure, the item ‘collaboration’ (mean = 1.84) was rated as most sufficient on average, with ‘close of meeting’ (mean = .71) rated as least sufficient on average. Pertaining to team formulation content, the item ‘consideration of significant life events’ (mean = 1.67) was rated as most sufficient on average, with the item ‘goals and values’ (mean = .64) rated as least sufficient on average across all team formulation sessions evaluated.

**TABLE 2:** Frequency of scoring for each TFQS item.

TFQS Item	Mean	SD	*N* = 0	*N* = 1	*N* = 2
A1 – Session Opening	.80	.66	15 (32.6%)	25 (54.3%)	6 (13%)
A2 – Collaboration	1.84	.42	1 (2.2%)	5 (10.9%)	40 (87%)
A3 – Interpersonal Effectiveness	1.33	.53	0 (0%)	31 (67.4%)	15 (32.6%)
A4 – Eliciting and Responding to Feedback	1.24	.53	3 (6.5%)	30 (65.2%)	13 (28.3%)
A5 – Summary Statements	1.40	.62	3 (6.5%)	22 (47.8%)	21 (45.7%)
A6 – Pacing	1.31	.70	7 (15.2%)	19 (41.3%)	20 (43.5%)
A7 – Close of Meeting	.71	.66	18 (39.1%)	22 (47.8%)	5 (10.9%)
Section A Total	8.44	2.34			
B1 – Description of service user	1.33	.60	3 (6.5%)	25 (54.3%)	18 (39.1%)
B2 – Problems identified	1.11	.32	1 (2.2%)	40 (87.0%)	5 (10.9%)
B3 – Strengths	1.07	.58	6 (13.0%)	31 (67.4%)	9 (19.6%)
B4 – Goals & Values	.64	.65	21 (45.7%)	21 (45.7%)	4 (8.7%)
B5 – Life Events	1.67	.48	0 (.0%)	16 (34.8%)	30 (65.2%)
B6 – Team Coping	1.16	.71	9 (19.6%)	22 (47.8%)	15 (32.6%)
B7 – Social and Cultural Factors	1.07	.58	7 (15.2%)	30 (65.2%)	9 (19.6%)
B8 – Support Plans	1.04	.42	3 (6.5%)	37 (80.4%)	5 (10.9%)
Section B Total	9.09	2.11			

### Aim 3: Evaluating the relationship between observer‐rated and self‐report TFQS ratings

A weak but statistically significant positive correlation (*n* = 39; *r* = .333; *p* = .039) was observed between the first author's independent ratings (*M =* 17.56, SD = 3.70) using the TFQS and the psychologists' own ratings (*M* = 22.83, SD = 3.82) of the quality of formulation sessions using an adapted version of the TFQS. Regarding the TFQS subscale A, a weak positive correlation (*n* = 39; *r* = .232; *p* = .156) was observed between the independent ratings (*M* = 8.38, SD = 2.52) using the TFQS and the psychologists' own ratings (*M* = 10.23, SD = 1.69) using the adapted version of the TFQS. A weak positive correlation (*n* = 39; *r* = .242; *p* = .138) was observed between the independent rating (*M* = 8.95, SD = 2.34) and the psychologists' own ratings (*M* = 11.85, SD = 3.06) for TFQS subscale B.

## DISCUSSION

This study aimed to investigate the reliability of the TFQS within acute mental health teams within a larger sample size than the original scale development study and without those involved in the original research team administering the tool. The study found that the TFQS demonstrated excellent inter‐rater reliability in this setting. A second aim was to provide descriptive data regarding team formulation practices in acute mental health settings. Relating to the structure of team formulation sessions, the study identified the TFQS item for ‘collaboration’ as rated highest on average in this setting, with the item relating ‘close of meeting’ rated as least sufficient on average. With regard to the content of team formulation sessions, the item relating to ‘consideration of life events’ was rated as most sufficient on average, with the item relating to consideration of a service user's ‘goals and values’ rated as least sufficient on average. An additional aim was to explore the relationship between observer‐rated and the team formulation facilitators' self‐reported ratings of team formulation quality using the TFQS. The study found a weak but statistically significant positive relationship between observer‐rated and facilitators' self‐reported ratings of team formulation quality.

The level of reliability found in this study (ICC = .926) was much higher than the level of reliability reported in the original study (ICC = .585). One possible explanation for this is that the two researchers that administered the TFQS in this study were trained together in the use of the TFQS prior to administering the scale independently, and supervision was used throughout the rating period to check understanding of items. An additional explanation may be that this study used a larger sample size. Whilst a larger size would only statistically lead to narrower confidence intervals rather than an increased correlation coefficient (Liljequist et al., 2019), the smaller sample size in Bucci et al.'s (2021) original study may have meant that any large discrepancies in individual cases could have had a larger impact on the overall agreement than in this study.

The reliability of individual TFQS items ranged from ‘fair’ to ‘substantial’ which was lower in comparison to the level of reliability of the overall scale and subscales of the TFQS. One possible reason for this difference may be due to overlap between TFQS items in which an aspect of team formulation could be considered relevant to multiple TFQS items. For example, the team formulation facilitator offering praise and acknowledging strengths in the approach of the team could be attributed to either of items A3 (interpersonal effectiveness) and B3 (strengths and resources). Similarly, a description of ‘positive’ (desirable) traits of the service user could be attributed to both items A1 (description of a service user) or B3 (strengths and resources). Raters may be less consistent in their ratings of individual items, though the information will still be accounted for within the overall scoring of the TFQS. However, to address the issue of reliability of items going forward, the TFQS may benefit from further specification in the manual regarding scoring of certain items and distinctions in overlap between items.

Regarding team formulation content, the TFQS items relating to consideration of ‘significant life events’ and ‘description of the service user’ were rated as most sufficient on average within the current sample. Other TFQS items relating to consideration of ‘goals and values’, ‘social and cultural factors’, ‘strengths’, and ‘support plans’ were rated as less sufficient on average. In considering these findings in relation to the wider literature, studies of team formulation practices have described increased understanding of a service user's difficulties as both an aim and outcome of team formulation (Bealey et al., 2021; Geach et al., 2018). However, in reviews of studies of team formulation practices, factors such as understanding of a person's goals, values, strengths, and resources appear absent from the definition, aims, and outcomes of team formulation practices (Bealey et al., 2021; Geach et al., 2018). In this sense, current approaches to team formulation may be largely problem focused with less emphasis on understanding a person's strengths and resources, goals and values, and important cultural factors.

This problem focus may reflect the absence of the service user's voice within team formulation. The function of team formulation is multifaceted and is thought to benefit both staff teams and service users. Consideration of the person's views and ideally their contribution to a formulation is recommended practice in individual and team formulation (ACP, 2022). Within this study, the psychologists facilitating team formulation sessions often acknowledged that discussions were hypotheses and at times attempted to recall service users' views expressed previously. However, whilst consideration of service user views is reflected in the scoring of some TFQS items, the current study did not directly measure service user involvement in team formulation practices nor the impact of their involvement on team formulation quality. In this study, greater emphasis on important cultural factors may have been achieved through increased service user involvement. The relative neglect of cultural factors is also reflective of wider issues faced particularly by ethnic minorities in which mental health services are perceived as unresponsive and insensitive to individual or cultural preferences (Bansal et al., 2022; Memon et al., 2016). Regarding team formulation structure, this study indicated that further consideration should be given to the opening and closing of team formulation sessions, with those respective TFQS items being rated as least sufficient on average in the current sample.

A weak correlation was found between the independent ratings using the TFQS and the psychologists' own ratings using an adapted version of the TFQS. Psychologists' ratings were on average more generous than observer ratings of team formulation quality (*M* = 22.83 vs. *M* = 17.48). Research has indicated that clinicians' self‐reported skill and fidelity to models of case formulation may be unreliable (Carroll et al., 1998; Creed et al., 2014; Hogue et al., 2013; Hurlbert et al., 2010; Kuyken et al., 2005; Martino et al., 2009; Zivor et al., 2013). The differences between clinicians' self‐appraisal and appraisals by observers in this study and in wider research emphasize the importance of observers' ratings when administering the TFQS. However, in clinical and training settings where an observer may not always be available, the TFQS may still be useful as a self‐checklist for clinicians facilitating team formulation practices.

### Recommendations for future research

The current study found the TFQS to be a reliable measure of the quality of team formulation in this setting; however, future research may investigate the reliability of the measure in other settings involving multidisciplinary teams. In this study, a compassion‐focused approach was used in 54% of team formulation sessions. It is also notable that one psychologist facilitated 29 (60.4%) of the team formulation sessions used in the analysis in this study, which presents issues with the generalization of the results of this study. The TFQS would benefit from further research exploring the validity and reliability of the tool with a wider pool of clinicians and approaches to formulation. The weak correlation between observer ratings and clinicians' self‐assessment of team formulation quality using the TFQS indicates that further research is needed to explore the validity of self‐rated team formulation quality when compared with independent ratings using the TFQS. Scoring of the TFQS is partly dependent upon clinicians' understanding of a service user's views. However, this study indicates that future research should directly explore the impact of service users' involvement in the development of team formulation and the impact on the quality of team formulation practices. It could be argued that a limitation of the TFQS is that the structure (sections A and B) is not empirically derived. The measure's structure is consistent with findings from a recent Delphi study exploring clinicians' views of the core components of team formulation (Thrower et al., 2024) and other measures used to assess the quality of case formulation in individual therapy (Bucci et al., 2016). However, future studies should conduct factor analysis to test the hypothesized subscales of ‘process’ and ‘content’ within the TFQS, which was not possible in the current study due to the small sample size. This future research is important as it could be argued that Section A of the scale reflects non‐specific factors akin to the non‐specifics within individual therapy, whereas Section B of the scale is more reflective of clinicians' skills in formulation. Although we would argue that the process skills captured within Section A reflect the capacity to orchestrate multiple viewpoints and assimilate this information in vivo, which is a core component of developing a good team formulation and a highly complex skill set to master.

### Clinical implications

To the authors' knowledge, the TFQS is the only tool developed to enable measurement of team formulation quality in mental health settings. With practices of team formulation becoming more common (ACP, 2022), the TFQS may be a useful tool for researchers and clinicians in evaluating team formulation practices. The TFQS also helps to address the need for greater standardization and specification in team formulation practices and research (Geach et al., 2018).

Descriptive data from this study indicated that a service user's strengths, goals, and values, and social and cultural factors lacked sufficient consideration overall in team formulation practices. Clinicians involved in team formulation practices may look to dedicate time for consideration of how these factors relate to support and care plans identified through team formulation practices. This could be supported through increased focus in these areas in clinical psychology training and by the development of published guidelines regarding the structure and content of team formulation. The Power Threat Meaning Framework (Johnson & Boyle, 2018) appears to be a suitable and relevant approach for supporting service users' perspectives and constructing a narrative around their experiences.

## CONCLUSION

The current study supports previous findings that the TFQS may be a reliable tool for measuring the quality of the structure and content of team formulation sessions in clinical and research settings. The study indicates that approaches to understanding and working with service users in mental health settings would benefit from further consideration of relevant social and cultural factors of the service users' experience, their goals, values, strengths, and resources, and consideration of these factors in developing appropriate support plans and interventions. There appears to be a weak positive relationship between psychologists' own ratings for the quality of team formulation practices and those of observers, indicating differences between self and observer ratings, which require further exploration through research.

## AUTHOR CONTRIBUTIONS

**Joshua Eli Thompson:** Conceptualization; writing – original draft; formal analysis; project administration; data curation. **Gillian Haddock:** Writing – review and editing; supervision. **Georgia Penn:** Writing – review and editing; project administration. **Katherine Berry:** Conceptualization; writing – review and editing; supervision.

## FUNDING INFORMATION

GH and KB are supported by the National Institute for Health Research (NIHR) Manchester Biomedical Research Centre (NIHR 203308). The views expressed are those of the authors and not necessarily those of the NIHR or the Department of Health and Social Care.

## CONFLICT OF INTEREST STATEMENT

None.

## Data Availability

The data and findings presented in this paper area part of a larger study reported in Thompson (2024) available via the University of Manchester Library. The original contributions presented in the study are included in the article/supplementary material; further inquiries can be directed to the corresponding author.
